# Prediction of clinical response to drugs in ovarian cancer using the chemotherapy resistance test (CTR-test)

**DOI:** 10.1186/s13048-017-0365-9

**Published:** 2017-10-27

**Authors:** Frank Christian Kischkel, Carina Meyer, Julia Eich, Mani Nassir, Monika Mentze, Ioana Braicu, Annette Kopp-Schneider, Jalid Sehouli

**Affiliations:** 1 TherapySelect, Heidelberg, Germany; 2 Charité Berlin, Gynecology Department, Virchow Campus Berlin, Berlin, Germany; 3 German Cancer Research Center, Division of Biostatistics, Heidelberg, Germany

**Keywords:** CTR-test, Individualized cancer therapy, Ovarian cancer, Sensitivity and resistance prediction, Resistance classification system

## Abstract

**Background:**

In order to validate if the test result of the Chemotherapy Resistance Test (CTR-Test) is able to predict the resistances or sensitivities of tumors in ovarian cancer patients to drugs, the CTR-Test result and the corresponding clinical response of individual patients were correlated retrospectively. Results were compared to previous recorded correlations.

**Methods:**

The CTR-Test was performed on tumor samples from 52 ovarian cancer patients for specific chemotherapeutic drugs. Patients were treated with monotherapies or drug combinations. Resistances were classified as extreme (ER), medium (MR) or slight (SR) resistance in the CTR-Test. Combination treatment resistances were transformed by a scoring system into these classifications.

**Results:**

Accurate sensitivity prediction was accomplished in 79% of the cases and accurate prediction of resistance in 100% of the cases in the total data set. The data set of single agent treatment and drug combination treatment were analyzed individually. Single agent treatment lead to an accurate sensitivity in 44% of the cases and the drug combination to 95% accuracy. The detection of resistances was in both cases to 100% correct. ROC curve analysis indicates that the CTR-Test result correlates with the clinical response, at least for the combination chemotherapy. Those values are similar or better than the values from a publication from 1990.

**Conclusions:**

Chemotherapy resistance testing in vitro via the CTR-Test is able to accurately detect resistances in ovarian cancer patients. These numbers confirm and even exceed results published in 1990. Better sensitivity detection might be caused by a higher percentage of drug combinations tested in 2012 compared to 1990. Our study confirms the functionality of the CTR-Test to plan an efficient chemotherapeutic treatment for ovarian cancer patients.

**Electronic supplementary material:**

The online version of this article (10.1186/s13048-017-0365-9) contains supplementary material, which is available to authorized users.

## Background

Ovarian cancer is one of the leading causes of cancer-related death in women. According to the Robert Koch-Institut, in 2012 in Germany 7380 women were newly diagnosed with ovarian cancer. Worldwide in 2012 there were about 239,000 new ovarian cancer patients (according to World Cancer Research Fund International). Numerous patients show advanced stages due to a late diagnosis. Therefore the prognosis is rather poor and the relative 5 year survival rate is only about 41% (Robert Koch-Institut, 2012). Furthermore, the recurrence rate is very high (around 70%) [1, 2]. The standard first-line treatment for ovarian cancer is cytoreductive surgery followed by a combination chemotherapy with carboplatin and paclitaxel [2–5].

However, several studies indicate, that tumor cells exhibit a different chemosensitivity to chemotherapeutics in distinct patients which influences the clinical outcome of a chemotherapy [6–11]. Therefore, standard treatments can be ineffective in single patients because of drug resistance of the tumor and a next-line therapy is needed due to a relapse. Thus, it is useful to identify in vitro the resistance profile of a set of chemotherapeutics which are considered for the individual patient, before a potential ineffective chemotherapy is applied [12]. Thereby, the therapeutic benefit can be increased, unnecessary toxicity is avoided, costs are reduced and valuable time is saved [7, 13].

In 1990 Kern and Weisenthal introduced a resistance classification system based on in vitro testing of living tumor material. They analyzed 450 tumor samples in vitro via the EDR (extreme drug resistance) assay to find the particular resistance of patients and compared those results to the actual treatment success of the individual patients [6]. The EDR assay shows a > 99% accuracy in detecting chemotherapeutics that are ineffective and do not result in a clinical benefit for an individual patient [6].

The CTR-Test®, which was employed in this paper, is identical to the EDR assay. The objective of this paper was to demonstrate functionality as well as usefulness of the CTR-Test regarding prediction of clinical response to drugs in ovarian cancer. We used 52 ovarian cancer samples and tested them via the CTR-Test to identify the drug resistance of the individual patients to specific chemotherapeutics applied as single drugs or in combination. The results from the CTR-Test were compared to the clinical response of the patients. Furthermore, our data were compared to the data from Kern and Weisenthal [6].

## Methods

### Patients

During the time span January 2010 – January 2011 96 female ovarian cancer patients were included in this study. Therefore, fresh tumor material from the patients was sent to TherapySelect from the Charité in Berlin within 24 h to perform the CTR-Test, which tests the drug resistance in vitro. A retrospective single-blinded study was performed. The treating physicians received the CTR-Test results before therapy, which could be used by the physicians for therapy planning. The company performing the CTR-Test was blinded to the response rate. The response rate of the used chemotherapeutic drug was compared to the result of the CTR-Test. Only 52 patients could be included in this study. The other patients were excluded because in 12 cases there was no chemotherapy treatment applied or it was not clear which drug was chosen. In 11 cases there was no CTR-Test result available (due to insufficient tumor material) and in 17 cases a chemotherapeutic drug was chosen which was not tested by the CTR-Test. In 4 cases no or no clear follow up was available. In the remaining cases the CTR-Test results were classified into resistance classes and the treatment success was defined.

### Resistance and treatment response classification

The CTR-Test determines the percent cell growth inhibition (PCI) values and those are classified into 3 different classes. These are: extreme resistance (ER) with a PCI ≤ mean – 1 SD, medium resistance (MR) with a PCI between mean and mean – 1 SD and slight resistance (SR) with a PCI ≥ mean. The mean and the standard deviation are determined by the PCI values of several samples treated with the chosen drug.

The patients were classified as responder or non-responder depending on the clinically evaluated treatment success. Treatment success was determined by physician’s evaluation. Physicians assessed disease outcomes at follow-up visits on the available clinical data including imaging techniques (like CT-scans), CA125 levels, physical examinations and medical records. The response classifications were extracted from the medical records as is. The responders are defined as clinical complete response or partial response, whereas the non-responders showed clinical stable disease or progressive disease.

### CTR-test

The CTR-Test was performed regarding published protocols [6, 14]. Following surgery the tumor samples were immediately immersed in incubation medium and sent promptly to laboratory. Tumor specimens were minced with sharp surgical scissors and then subjected to enzymatic digestion using 0.14% collagenase type I and 0.01% DNAse. The resulting small aggregates (spheroids) and single cells were centrifuged and the pellet was suspended in 5 ml of minimum-essential tissue culture medium containing heat-inactivated fetal calf serum, 100 units of penicillin, 100 μg/ml of streptomycin and 200 mM L-glutamine. Tumor cell percentage and cell viability was assessed by an external pathology. The cell viability was between 50% - > 90% and the tumor cell content was between 40% - 90% (see Additional file 1). Soft agar matrix (0.4%) selective for tumor growth was placed in 24-well polystyrene culture dishes and 50 μl of one specific chemotherapeutic drug was then added to appropriate wells. The drug concentrations can be found in [15]. Tumor cells suspended in 0.5 ml tissue culture medium and 0.2% agarose were then added to each well. After 72 h incubation at 37 °C in the presence of 5% CO2, 5.0 μCi H-3-thymidine was introduced into each well, and the plates were incubated for an additional 48 h to allow radioactive thymidine incorporation into the DNA of the surviving tumor cells. Cellular DNA was collected on glass-fiber filters using a PerkinElmer harvester. The isotope uptake and the corresponding DNA amount is determined by scintillation counting. The data obtained are counts per minute (cpm). Treating cells with a lethal dose of a drug is used as a positive control and no addition of drugs to cells is used as negative control. The percent cell growth inhibition (PCI) value represents the effectiveness of the chemotherapeutic. PCI values are calculated using the formula: PCI = (cpm(treated cells)-cpm(positive control))/(cpm(negative control)-cpm(positive control)).

### Drugs used for analysis

The to be tested drugs in this study were chosen after therapeutic relevance and validated before used in the CTR-Test. Validation was used to find concentrations in a physiological range and it was performed with tumor samples to set the concentration to a level which leads to a satisfying distribution of drug action among a collective of samples.

### Statistical analysis

Receiver operator characteristic (ROC) curve analysis was conducted in R. The 95% confidence intervals for proportions were determined by the Clopper-Pearson method.

## Results

From 2010 to beginning of 2011 96 ovarian cancer patients from the Charité Berlin participated in this study concerning the retrospective diagnostic capacity of the CTR-Test. Patients were treated regarding the standard guidelines (according to the Association of the Scientific Medical Societies in Germany) and tumor samples were analyzed with the CTR-Test to determine the in vitro resistance of the tumor cells towards the selected chemotherapeutic drugs. From those 96 patients, 52 data sets were suitable to be included in this analysis because in those cases the CTR-Test presented a result and the patients received a chemotherapy with a single drug or drug combination tested by the CTR-Test (Table 1). Forty-four patients had to be excluded from the study due to several reasons, which can be found in Table 1 and in the Methods part of this paper.

**Table 1:** Classification of 96 ovarian cancer patients regarding their analyzability for the study

Number	Analyzability	Explanation
52	Analyzable	Patients received a traceable chemotherapy and the used drugs were analyzed by the CTR-Test
44	Not analyzable	Patients either did not receive a chemotherapy (12), drugs were used that were not measured in the CTR-Test (17), the CTR-Test did not yield a result (11) or no record about chemotherapy or response is available (4)

The remaining 52 patients were treated either with monotherapies (19 patients) or combination therapies (33 patients). In the monotherapy group, 3 patients were treated additionally with Avastin (Table 2).

**Table 2:** Classification of 52 analyzable ovarian cancer patients (see Table 1) into three groups regarding the applied therapy

Number	Group name	Description of therapy
16	Mono	Patients received a monotherapy with one chemotherapeutic drug
3	(Mono)	Patients received a monotherapy with one chemotherapeutic drug plus a treatment with Avastin
33	Combi	Patients received a combination therapy with 2 drugs

For each included patient the applied chemotherapy and the corresponding CTR-Test result were documented. Additionally, the chemotherapy success was determined by physician’s evaluation and patients were classified accordingly as responder or non-responder. In Table 3 the patients are separated in responder or non-responder and the used chemotherapeutics are listed with the corresponding CTR-Test results. Furthermore, the different therapy types (monotherapy, monotherapy plus Avastin, combination therapy) are analyzed individually (Table 3). According to former publications [6–11], we defined three resistance categories based on the percent cell growth inhibition (PCI) values of the patients and the responses were scored. For each drug a heterogeneous patient population was analyzed for their response to that particular drug. ER (extreme resistance) is characterized by PCI < mean – 1SD (standard deviation) and is scored “0”. PCI values ≥ mean are defined as SR (slight resistance) and are scored “2”. MR is classified as PCI > ER but < mean and is scored “1”.

**Table 3:** Clinical response of individual patients after monotherapy, monotherapy plus Avastin or combination therapy

Non-Responder				Responder			
Patient	Substance	CTR-Test		Patient	Substance	CTR-Test	
Monotherapy							
1	Carbo	ER		13	Pac	SR	
2	Caelyx	SR		14	Carbo	SR	
3	Topo	SR		15	Topo	SR	
4	Carbo	SR		16	Pac	MR	
5	Topo	MR					
6	Topo	MR					
7	Caelyx	MR					
8	Caelyx	ER					
9	Caelyx	MR					
10	Topo	SR					
11	Topo	ER					
12	Topo	ER					
(Mono)							
1	Caelyx + Avastin	SR		2	Caelyx + Avastin	SR	
				3	Topo + Avastin	MR	
Combination Chemotherapy							
Patient	Substance	CTR-Test	Score^a^	Patient	Substance	CTR-Test	Score^a^
1	Carbo/Pac	ER/ER	0	9	Carbo/Caelyx	SR/SR	4
2	Carbo/Caelyx	MR/ER	1	10	Carbo/Gem	SR/SR	4
3	Carbo/Gem	ER/ER	0	11	Carbo/Pac	SR/SR	4
4	Carbo/Pac	SR/SR	4	12	Carbo/Caelyx	SR/ER	2
5	Carbo/Gem	ER/ER	0	13	Carbo/Caelyx	SR/ER	2
6	Carbo/Pac	ER/SR	2	14	Carbo/Caelyx	SR/MR	3
7	Carbo/Caelyx	MR/ER	1	15	Carbo/Caelyx	SR/SR	4
8	Carbo/Pac	MR/ER	1	16	Carbo/Pac	MR/ER	1
				17	Carbo/Pac	SR/MR	3
				18	Carbo/Caelyx	MR/SR	3
				19	Carbo/Caelyx	SR/SR	4
				20	Carbo/Pac	SR/MR	3
				21	Carbo/Caelyx	SR/MR	3
				22	Carbo/Gem	ER/SR	2
				23	Carbo/Pac	SR/SR	4
				24	Carbo/Caelyx	MR/MR	2
				25	Carbo/Pac	SR/MR	3
				26	Carbo/Caelyx	MR/MR	2
				27	Carbo/Caelyx	SR/MR	3
				28	Carbo/Pac	SR/MR	3
				29	Carbo/Pac	SR/MR	3
				30	Carbo/Pac	SR/SR	4
				31	Carbo/Pac	SR/SR	4
				32	Carbo/Pac	ER/SR	2
				33	Carbo/Caelyx	SR/SR	4

The applied chemotherapeutics and the corresponding results of the CTR-Test for individual patients are listed. Patients were classified as non-responders and responders

*Abbreviations*: *Carbo* Carboplatin, *Gem* Gemcitabine, *Pac* Paclitaxel, *Topo* Topotecan, *ER* extreme resistance, *MR* medium resistance, *SR* slight resistance, *Caelyx* doxorubicin-hydrochloride in a pegylated liposomal formulation

^a^For combination therapy the two single drugs were measured and scores were determined according to the resistance categories: ER = 0; MR = 1; SR = 2. The scores obtained are 4 = SR/SR; 3 = MR/SR, SR/MR; 2 = ER/SR, SR/ER, MR/MR; 1 = ER/MR, MR/ER; 0 = ER/ER

These classifications are comparable with the EDR, IDR and LDR classes introduced in 1990 by Kern and Weisenthal [6]. To predict the effect of drug combinations, the two corresponding single measurements are combined to define the resistance class of the combination. A scoring system, introduced by [7], is used for predicting the grade of resistance of a combination based on the single measurements. Therefore the single resistance of ER is scored with 0, MR with 1 and SR with 2 and the combination score is accomplished by summing up both single scores. This creates a range with a maximum of 4 and a minimum of 0 [7] (Table 3). To transfer the combination scores back to the resistances classes, score 4 and 3 are combined to create the class of SR. Two and one represent the class of MR and 0 is equal to ER.

The distribution of CTR-Test resistance classes for responder and non-responder was determined and compared with each other. This was done separately for monotherapy (Mono), monotherapy plus Avastin ((Mono)) and the combination therapy (Combi) data set. To get a general picture, the two monotherapy data sets (Mono/(Mono)) were combined and then united with the combination data set.

### Comparison of Charité data to Kern and Weisenthal paper

Kern and Weisenthal [6] analyzed the CTR-Test resistance profile of 450 samples from patients with a wide range of tumor types and compared those with the actual response rates of chemotherapies in patients. With defining the extreme resistance (EDR) class, an accuracy of 99% was achieved in predicting inefficient chemotherapies correctly (95% CI: 0–4). In 52% of the cases (95% CI: 45–59) the prediction of sensitivity (LDR) was correct, meaning that the patients did respond to the chosen chemotherapy and the CTR-Test predicted this outcome (Table 4) [6]. In contrast to Kern and Weisenthal [6] we only focused on ovarian cancer. Looking at the total data set from the Charité Berlin from 2012, the values for sensitivity and resistance outnumber the total data set values from 1990 with a sensitivity precision of 79% for SR (22 responder from 28) (95% CI: 59–92) and an ER resistance detection of 100% (0 responder from 7) (95% CI: 0–41) (Table 4). The values for the medium resistance are 16% (95% CI: 9–24) accuracy predicting sensitivity and on the other hand 84% accuracy for predicting resistances in case of Kern and Weisenthal (Table 4) [6]. The Charité values for MR are to 53% accurate in sensitivity prediction and to 47% accurate in resistance prediction (95% CI: 28–77) (Table 4).

**Table 4:** Correlation of CTR-Test results and clinical response and comparison to Kern and Weisenthal data

	SR		MR		ER	
	Resp. / n	% 95% CI	Resp. / n	% 95% CI	Resp. / n	% 95% CI
Total data set (Ovarian cancer) Charité Berlin 2012	22/28	79 59–92	9/17	53 28–77	0/7	0 0–41
Total data set Kern DH, Weisenthal LM (1990)	115/222	52 45–59	16/101	16 9–24	1/127	1 0–4
Ovarian cancer Kern DH, Weisenthal LM (1990)	14/24	58 37–78	0/11	0 0–28	0/11	0 0–28
Single agent chemotherapy Kern DH, Weisenthal LM (1990)	72/151	48 40–56	9/79	16 5–21	1/115	1 0–5
Single agent chemotherapy Charité Berlin 2012	4/9	44 14–79	2/6	33 4–78	0/4	0 0–60
Combination chemotherapy Kern DH, Weisenthal LM (1990)	43/71	61 48–72	7/22	32 14–55	0/12	0 0–26
Combination chemotherapy Charité Berlin 2012	18/19	95 74–100	7/11	64 31–89	0/3	0 0–71

The number of responders (Resp.) among the total number of patients (n) for the three different CTR-Test results are listed. The percentage and the 95% confidence interval (CI) are indicated

From Kern and Weisenthal 1990, the total data set, ovarian cancer, single agent and combination chemotherapy data are presented. From the Charité Berlin 2012 (see Table 3), the total data set, single agent and combination chemotherapy data are presented

Kern and Weisenthal separated their data set into subsets for each cancer type which offers the possibility to directly compare the response distribution in the setting of ovarian cancer (No. of patients 46) with the data set of patients participating in the Charité study (No. of patients 52) (Table 4). For ovarian cancer in 1990, an accuracy of 100% for resistance in the EDR class (95% CI: 0–28) (100% for the 2012 study, 95% CI: 0–41) and a 58% accuracy in predicting sensitivity in the LDR class (95% CI: 37–78) (79% for the 2012 study, 95% CI: 59–92) was reached (Table 4).

The 2012 data set can be separated into distinct groups of single agent and combination treatment. Both sets are compared to data from the Kern and Weisenthal paper [6]. For single agent treatment, values are measured of 44% SR sensitivity (95% CI: 14–79) (Table 4) compared to 48% in 1990 (95% CI: 40–56) (Table 4), MR sensitivity of 33% (95% CI: 4–78) (Table 4) compared to 16% (95% CI: 5–21) (Table 4) and ER resistance of 100% (95% CI: 0–60) (Table 4) and 99% (95% CI: 0–5), respectively (Table 4). The single agent analysis is in our case ovarian cancer specific and consists of 19 different patients. In comparison, Kern and Weisenthal analyzed 345 samples of different tumor types [6].

The second subset is comprised of data derived from tumor samples treated with a drug combination. Here, in 2012 values of 95% SR sensitivity (95% CI: 74–100), 64% MR sensitivity (95% CI: 31–89) and 100% ER resistance (95% CI: 0–71) was measured (Table 4) which is confronted with 1990 values of 61% (95% CI: 48–72), 32% (95% CI: 14–55) and 100% (95% CI: 0–26), respectively (Table 4). In the 2012 combination subset 33 samples were included and in 1990 105 samples.

**Fig. 1:**
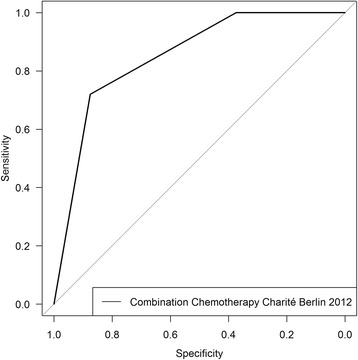
Receiver Operating Characteristic (ROC) curve analysis for the combination chemotherapy. Tumor samples from 33 ovarian cancer patients (Charité Berlin, 2012) were used. A CTR-Test was performed and the patients were treated with a combination chemotherapy which was measured by the CTR-Test. The individual patients were evaluated regarding their clinical response (responder or non-responder) and their CTR-Test result (SR, MR or ER) (see Table 3). A ROC curve analysis was performed to investigate whether the CTR-Test is able to predict the clinical response. The corresponding area under the curve (AUC) value is 0.85 and the 95% confidence Interval (CI) is 0.6942–1

**Fig. 2:**
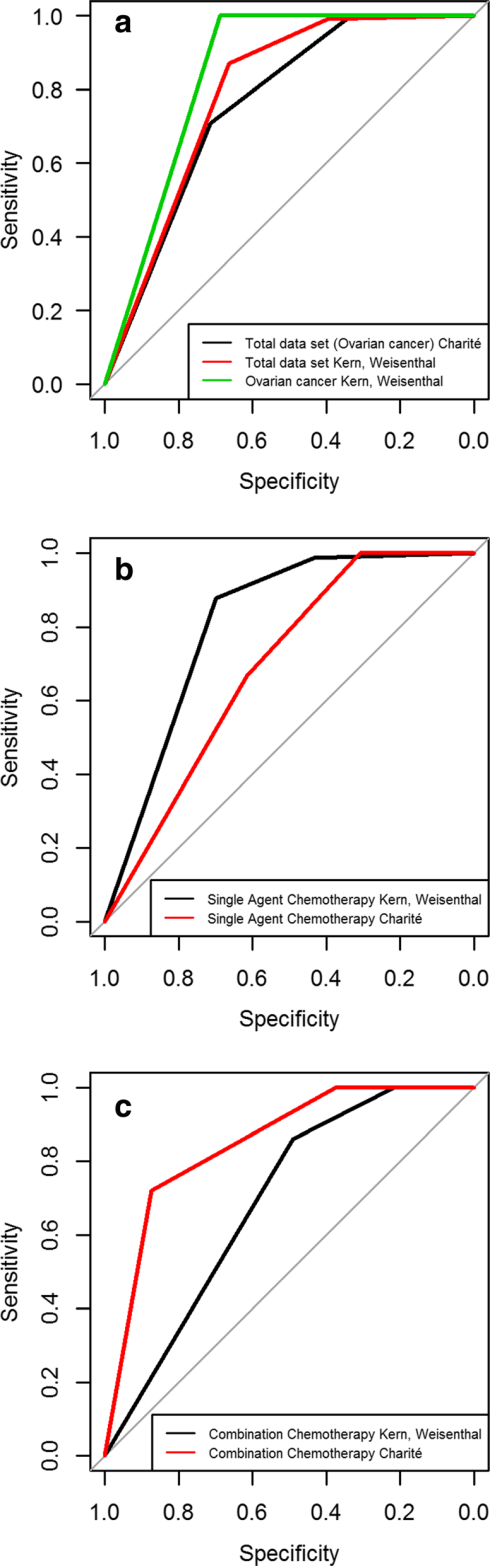
Receiver Operating Characteristic (ROC) curve analysis for the total data set, the single chemotherapy, the combination chemotherapy and the data sets from Kern and Weisenthal**. a** Tumor samples from 52 ovarian cancer patients (total data set Charité Berlin, 2012) were used. A CTR-Test was performed and patients were treated with a monotherapy, a monotherapy plus Avastin or a combination chemotherapy which was tested by the CTR-Test. The individual patients were evaluated regarding their clinical response (responder or non-responder) and their CTR-Test result (SR, MR or ER) (see Table 3). A ROC curve analysis was performed for our total data set and for data from Kern and Weisenthal 1990 (total data set and ovarian cancer) to investigate whether the CTR-Test is able to predict the clinical response. AUC total data set Charité Berlin 2012: 0.7604; 95% CI: 0.6328–0.888. AUC total data set Kern and Weisenthal 1990: 0.7904; 95% CI: 0.7565–0.8242. AUC ovarian cancer Kern and Weisenthal 1990: 0.8438; 95% CI: 0.7622–0.9253. **b** Tumor samples from 19 ovarian cancer patients (Charité Berlin, 2012) were used. A CTR-Test was performed and the patients were treated with a monotherapy or a monotherapy plus Avastin. The monotherapy was tested by the CTR-Test. Patients were evaluated as described above. A ROC curve analysis was performed for our data and for data from Kern and Weisenthal 1990 (single agent chemotherapy). AUC single agent chemotherapy Kern and Weisenthal 1990: 0.811; 95% CI: 0.7728–0.8492. AUC single agent chemotherapy Charité Berlin 2012: 0.6923; 95% CI: 0.4741–0.9105. **c** Tumor samples from 33 ovarian cancer patients (Charité Berlin, 2012) were used. A CTR-Test was performed and the patients were treated with a combination chemotherapy which was tested by the CTR-Test. The patients were evaluated as described above. A ROC curve analysis was performed for our data and for data from Kern and Weisenthal 1990 (combination chemotherapy). AUC combination chemotherapy Kern and Weisenthal 1990: 0.6907; 95% CI: 0.6101–0.7713. AUC combination chemotherapy Charité Berlin 2012: 0.85; 95% CI: 0.6942–1

A receiver operator characteristic (ROC) curve analysis for all above mentioned data sets (see Table 4) was performed to confirm the assumption that the CTR-Test is able to predict the clinical response of patients to a chemotherapy treatment (Figs. 1 and 2). As before, our data were compared to data from [6]. This ROC curve analysis revealed a significant correlation between the CTR-Test result and the clinical outcome in the case of combination chemotherapy (Fig. 1), indicated by the good area under the curve (AUC) value of 0.85 (95% CI: 0.6942–1).

In Fig. 2a the ROC curves of the total data set from the Charité Berlin 2012 (black curve), the total data set from [6] (red curve) and the ovarian cancer data from [6] (green curve) are compared. All three data sets show good AUC values and a correlation between test result and response. The AUC for the total data set Charité Berlin 2012 is 0.7604 (95% CI: 0.6328–0.888); the AUC for the total data set Kern and Weisenthal 1990 is 0.7904 (95% CI: 0.7565–0.8242); the AUC for the ovarian cancer data set Kern and Weisenthal 1990 is 0.8438 (95% CI: 0.7622–0.9253).

Figure 2b compares the ROC curves of the single agent chemotherapy from the Charité Berlin 2012 (red curve) and from Kern and Weisenthal 1990 (black curve). In contrast to the combination data, the total data set and the single agent chemotherapy data from Kern and Weisenthal 1990 (AUC: 0.811, 95% CI: 0.7728–0.8492), the single agent chemotherapy data from the Charité Berlin do not show a significant correlation between CTR-Test result and response of the patients (AUC: 0.6923, 95% CI: 0.4741–0.9105).

As mentioned above, the combination chemotherapy data from the Charité Berlin 2012 show a significant correlation between the CTR-Test result and the clinical outcome of the patients (AUC: 0.85, 95% CI: 0.6942–1) (Figs. 1, 2c, red curve). The combination chemotherapy data from Kern and Weisenthal 1990 also show a good AUC of 0.6907 (95% CI: 0.6101–0.7713) (Fig. 2c black curve). In the case of combination chemotherapy, our test performs better than the test from [6].

## Discussion

Our performed study showed a predictive power of the CTR-Test to detect resistances and sensitivities of ovarian cancer samples from patients treated at the Charité Berlin to chemotherapeutics prior to chemotherapy. Sensitivity and resistance is classified via a method introduced in 1990 by Kern and Weisenthal [6]. They analyzed 450 different tumor samples with the EDR assay (here called CTR-Test) and accomplished a correct sensitivity detection in 52% (95% CI: 45–59) of the cases when the EDR assay classified a drug as LDR/SR. It actually had a positive effect for the patient and was therefore classified as responder. The prediction of resistances was even more accurate with a 99% (95% CI: 0–4) success rate in the EDR/ER class of detecting correctly non-responders (Table 4) [6]. In comparison to those numbers, our newly performed retrospective study for ovarian cancer at the Charité accomplished a sensitivity rate of 79% (95% CI: 59–92) and a resistance detection of 100% (95% CI: 0–41) (Table 4). Additionally, a medium resistance class was introduced in 1990 and within this class Kern and Weisenthal detected correctly in 16% (95% CI: 9–24) of the cases sensitive substances (Table 4) [6]. In the data set from the Charité the success rate was 53% (95% CI: 28–77) (Table 4). Based on the 1990 data, a drug with a medium resistance was more likely to produce no effect in treating the tested tumor in the patient. Therefore the drug suggestion would be against such a drug and only drugs from the SR class should have been recommended. However, based on the new data from the Charité such a classified drug has a 53% chance to have an effect. In this case it is less likely that a drug from the MR class has no effect. However in both cases it might be better to exclude them because a not given but effective drug has less severe consequences than an administrated drug with no effect.

The so far presented comparison is between the total data sets of both studies. In case of the 1990 study several different tumor types are included. We only used samples from ovarian cancer patients. Therefore, it is useful to also compare the Charité data with the ovarian cancer data set from Kern and Weisenthal. For this subset a sensitivity in the SR class of 58% (95% CI: 37–78) was measured, which is slightly closer to the Charité value of 79%. The ER prediction is in both cases in 100% (95% CI Charité: 0–41; 95% CI Kern and Weisenthal: 0–28) of the cases correct. However, the MR discrepancies are remaining and even became more extreme (0% (95% CI: 0–28) to 53% (95% CI: 28–77)), making a clear prediction for MR drugs quite complicated (Table 4) [6]. However, in the ovarian cancer data from Kern and Weisenthal it is not clear how high the percentage of single drug agents compared to combination treatment is [6].

It seems that the ratio of single drug to combination treatment is a determining factor for the accuracy of the total data set. This conclusion occurs by looking at the combination and single drug treatment individually. Better sensitivity detection might be caused by a higher percentage of drug combinations tested in 2012 compared to 1990. For the single agent correlation the data set of Kern and Weisenthal [6] is quite similar to the data set from the Charité (Table 4). The accuracy for SR sensitivity is at 48% (95% CI: 40–56) (Table 4) and 44% (95% CI: 14–79) (Table 4), respectively. Looking at the correlation for the combination, a higher percentage is measured in both cases with 61% (95% CI: 48–72) (Table 4, SR for combination, 1990) compared to 48% (95% CI: 40–56) (Table 4, SR for single drugs, 1990) and 95% (95% CI: 74–100) (Table 4, SR for combination, Charité 2012) to 44% (95% CI: 14–79, SR for single drugs, Charité 2012) (Table 4) in the SR class. The same is seen for the MR determination, the sensitivity is to 32% (95% CI: 14–55) accurate for the combination in 1990 (Table 4) compared to the single agent with a percentage of 16% (95% CI: 5–21) (Table 4) [6]. In 2012 for MR the accuracy is in general higher but the trend is the same with 64% (95% CI: 31–89) (Table 4) for the combination and 33% (95% CI: 4–78) (Table 4) for the single agent therapy.

Here in this paper we measured the two individual drugs of a drug combination separately. We added the two scores of the single measurements to obtain the score and the resistance class of the combination. We recently published a paper where we described an in vitro system to predict the chemotherapeutic efficacy of drug combinations [15]. With this system it is possible to use single measurements to predict the efficacy of drug combinations in certain cases and to measure combinations directly. However, this system was not yet available when the samples for this paper were analyzed. By measuring the drug combinations directly with our in vitro system our data could have probably been improved.

A ROC curve analysis confirmed our assumption that the CTR-Test is able to predict the clinical response of a patient to a chemotherapy. Our data are comparable to the data from [6]. In the case of combination chemotherapy, our data even exceed their data. Treatment with a drug combination leads to a reduced resistance risk. The two used chemotherapeutics normally attack different target mechanism and therefore, the chances of developing a resistance is reduced [16–19].

All data sets from [6] show good AUC values and therefore their test result correlates with the clinical response. The total data set and the combination chemotherapy data set from the Charité Berlin 2012 also indicate a correlation between CTR-Test result and clinical response. The single chemotherapy data from the Charité do not show a significant correlation, which could be explained by a too small number of patients that received a monotherapy.

## Conclusions

In conclusion, our study in 2012 confirms and exceeds the results obtained in 1990. Important to note is that this study was performed only with 52 ovarian cancer samples and it is a retrospective study. To reinforce these findings a prospective study with a bigger sample size should be performed. Nevertheless, this study is in line with several different performed studies, suggesting the use of the CTR-Test as a suitable diagnostic test to find the right treatment plan for ovarian cancer and other tumor types.

## Additional file

Additional file 1: Percentage of viable cells and tumor cells. The percentage of viable cells and tumor cells of the different patient samples (Responder or Non-Responder for Monotherapy, Monotherapy plus Avastin or Combination Chemotherapy) is presented. (PDF 29 kb)

## Abbreviations

CTR-Test: Chemotherapy Resistance Test
EDR: Extreme drug resistance
ER: Extreme resistance
MR: Medium resistance
PCI: Percent cell growth inhibition
SR: Slight resistance
