# Supplementary material for: Prediction of clinical response to drugs in ovarian cancer using the chemotherapy resistance test (CTR-test)
Source: J Ovarian Res. 2017 Oct 27;10:72. doi: 10.1186/s13048-017-0365-9 (PMC5658930; doi:10.1186/s13048-017-0365-9)
Supplement: Additional file 1: — Percentage of viable cells and tumor cells. The percentage of viable cells and tumor cells of the different patient samples (Responder or Non-Responder for Monotherapy, Monotherapy plus Avastin or Combination Chemotherapy) is presented. (PDF 29 kb) [file 13048_2017_365_MOESM1_ESM.pdf]

Monotherapy

Non-Responder

TherapySelect ID	Patient	Substance	CTR-Test result	Percentage of viable cells [%]	Percentage of tumor cells [%]
TS00427	1	Carbo	ER	>90%	80%
TS00429	2	Caelyx	SR	>80%	80%
TS00442	3	Topo	SR	80%	90%
TS00527	4	Carbo	SR	85%	90%
TS00542	5	Topo	MR	80%	90%
TS00584/E008.102	6	Topo	MR	80%	90%
TS00601	7	Caelyx	MR	90%	90%
TS00654	8	Caelyx	ER	85%	80%
TS00684	9	Caelyx	MR	50%	90%
TS00701	10	Topo	SR	90%	70%
TS00720	11	Topo	ER	60%	50%
TS00581	12	Topo	ER	80%	90%

Responder

TherapySelect ID	Patient	Substance	CTR-Test result	Percentage of viable cells [%]	Percentage of tumor cells [%]
TS00507	13	Pac	SR	95%	90%
TS00560	14	Carbo	SR	60%	60%
TS00752	15	Topo	SR	90%	90%
TS00585	16	Pac	MR	80%	90%

(Mono)

Non-Responder

TherapySelect ID	Patient	Substance	CTR-Test result	Percentage of viable cells [%]	Percentage of tumor cells [%]
TS00763	1	Caelyx + Avastin	SR	90%	not known

Responder

TherapySelect ID	Patient	Substance	CTR-Test result	Percentage of viable cells [%]	Percentage of tumor cells [%]
TS00509	2	Caelyx + Avastin	SR	80%	80%
TS00580	3	Topo + Avastin	MR	80%	90%

Combination Chemotherapy

Non-Responder

TherapySelect ID	Patient	Substance	CTR-Test result	Percentage of viable cells [%]	Percentage of tumor cells [%]
TS00525_E008.092	1	Carbo/Pac	ER/ER	70%	85%
TS00557	2	Carbo/Caelyx	MR/ER	90%	80%
TS00597	3	Carbo/Gem	ER/ER	70%	80%
TS00639	4	Carbo/Pac	SR/SR	70%	50%
TS00698	5	Carbo/Gem	ER/ER	50%	40%
E026.007	6	Carbo/Pac	ER/SR	90%	90%
TS00796	7	Carbo/Caelyx	MR/ER	60%	80%
TS00724	8	Carbo/Pac	MR/ER	80%	90%

Responder

TherapySelect ID	Patient	Substance	CTR-Test result	Percentage of viable cells [%]	Percentage of tumor cells [%]
TS00446	9	Carbo/Caelyx	SR/SR	80%	70%
TS00447	10	Carbo/Gem	SR/SR	80-90%	90%
TS00505	11	Carbo/Pac	SR/SR	70%	85%
TS00535	12	Carbo/Caelyx	SR/ER	80%	90%
TS00558	13	Carbo/Caelyx	SR/ER	80%	80%
TS00587	14	Carbo/Caelyx	SR/MR	80%	60%
TS00644	15	Carbo/Caelyx	SR/SR	90%	80%
TS00650	16	Carbo/Pac	MR/ER	90%	80%
TS00653	17	Carbo/Pac	SR/MR	70%	80%
TS00660	18	Carbo/Caelyx	MR/SR	90%	60%
TS00661_E008.10(	19	Carbo/Caelyx	SR/SR	90%	95%
TS00676	20	Carbo/Pac	SR/MR	80%	90%
TS00680	21	Carbo/Caelyx	SR/MR	70%	90%
TS00699	22	Carbo/Gem	ER/SR	80%	70%
TS00711	23	Carbo/Pac	SR/SR	70%	90%
TS00745	24	Carbo/Caelyx	MR/MR	90%	80%
E026.003	25	Carbo/Pac	SR/MR	not known	not known
TS00748	26	Carbo/Caelyx	MR/MR	80%	90%
TS00750	27	Carbo/Caelyx	SR/MR	90%	90%
E026.004	28	Carbo/Pac	SR/MR	not known	not known
E026.005	29	Carbo/Pac	SR/MR	not known	not known
TS00759	30	Carbo/Pac	SR/SR	90%	80%
E026.006	31	Carbo/Pac	SR/SR	90%	80%
TS00797	32	Carbo/Pac	ER/SR	70%	80%
TS00747	33	Carbo/Caelyx	SR/SR	90%	75%
